# Magnetized plasma rotator for relativistic mid-infrared pulses via frequency-variable Faraday rotation

**DOI:** 10.1038/s41377-025-02047-x

**Published:** 2026-01-02

**Authors:** Dong-Ao Li, Guo-Bo Zhang, Francesco Pegoraro, Qian Zhao, Wen-Jun Liu, Xing-Long Zhu, De-Bin Zou, Jian-Xing Li, Alexander Pukhov, Zheng-Ming Sheng, Tong-Pu Yu

**Affiliations:** 1 College of Science, National University of Defense Technology, Changsha, 410073 China; 2 Physics Department, University of Pisa, Pisa, 56127 Italy; 3 CNR, Istituto Nazionale di Ottica (INO), Pisa, 56127 Italy; 4 Ministry of Education Key Laboratory for Nonequilibrium Synthesis and Modulation of Condensed Matter, Shaanxi Province Key Laboratory of Quantum Information and Quantum Optoelectronic Devices, School of Physics, Xi’an Jiaotong University, Xi’an, 710049 China; 5 Institute for Fusion Theory and Simulation, School of Physics, Zhejiang University, Hangzhou, 310058 China; 6 Institut für Theoretische Physik I, Heinrich-Heine-Universität Düsseldorf, Düsseldorf, 40225 Germany; 7 Key Laboratory for Laser Plasmas (MOE), School of Physics and Astronomy, Shanghai Jiao Tong University, Shanghai, 200240 China; 8 Collaborative Innovation Center of IFSA, Shanghai Jiao Tong University, Shanghai, 200240 China; 9 Tsung-Dao Lee Institute, Shanghai Jiao Tong University, Shanghai, 201210 China

**Keywords:** Magneto-optics, Plasma physics, Nonlinear optics, Mid-infrared photonics

## Abstract

Optical rotators based on the Faraday effect have been widely used in optical systems, such as optical isolation and circulators. However, due to the limitation of crystals, the application of such optical rotators in high-power lasers has been severely hindered. Here, we propose a novel plasma rotator based on the frequency-variable Faraday rotation (FVFR) in a compact manner, achieved by driving the magnetized underdense plasma with a relativistic linearly polarized laser. In the magnetized plasma, the drive laser undergoes photon deceleration and relativistic Faraday rotation, leading to the generation of relativistic polarization-tunable mid-infrared (mid-IR) pulse with intensity $\ge {10}^{16}$ W cm^−2^ and a spectral width of 5–25 μm. With different magnetic fields, the polarization angle of the generated mid-IR pulse can be well controlled. Especially, one can obtain a circularly polarized mid-IR pulse with the spatial average polarization degree of $\ge 0.94$ at a suitable external magnetic field. The robustness of the rotator has been well demonstrated through comprehensive three-dimensional particle-in-cell simulations across a wide range of laser and plasma parameters. Such a rotator via FVFR is valid from mid to far-infrared and even THz waveband, offering new opportunities for strong-field physics, attosecond science, laboratory astrophysics, etc, and paving the way for relativistic plasma magneto-optics and future relativistic plasma optical devices.

## Abstract (web summary)

A novel magnetized plasma rotator based on Frequency-Variable Faraday Rotation (FVFR) in the nonlinear plasma wake is put forward, which can manipulate efficiently the polarization of relativistic mid to far-infrared optical pulses and even THz waves.

## Graphical abstract

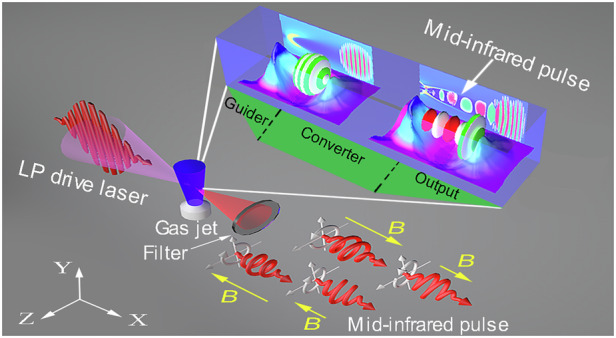

## Introduction

Faraday rotation (FR) or Faraday effect is a key phenomenon in magneto-optics^1^. It is prevalent in magnetized media and is the basis of optical isolation and rotator^2–5^, magnetic field sensor^6–8^, and current sensor^9^, etc. However, due to the limitation of the crystal itself, e.g., the heat effect and damage threshold, the magneto-active media is difficult to be miniaturized and integrated, as well as serve as a rotator for high-power lasers^10^. Plasma, as a kind of new medium without the damage threshold, allows for the fast propagation and modulation of high-power lasers. As a collection of charged particles, magnetized plasmas often give rise to a strong FR^11–13^, offering an important way to modulate the laser polarization. In recent years, the fast modulation and manipulation of polarization of high-power lasers in the near-infrared band in plasmas have made dramatic progress^11,14–18^. However, these methods mainly operate in non-relativistic cases without nonlinear optical effects, albeit with a very high polarization conversion efficiency. For example, the polarization state of a probe laser with intensity of 10^14 ^W cm^−2^ can be controlled by the beat wave^14^. In a plasma photonic crystal created in underdense plasma by counterpropagating laser beams, the laser pulse with intensity of 10^16 ^W cm^−2^ can be changed from linear to circular polarization, and the total energy transmission is close to 95% of the incident energy^16^. In an extreme FR case, a circularly polarized (CP) laser pulse with power as high as 5 PW can be generated by splitting a linearly polarized (LP) laser pulse with intensity of 10^16 ^W cm^−2^ in magnetized plasmas^11^.

The physical picture alters significantly in relativistic regime, i.e., $I{\left({\lambda }_{0}/{\rm{\mu }}{\rm{m}}\right)}^{2}\ge 1.37\times {10}^{18}$ W cm^−2^ ($I$ is the laser intensity and ${\lambda }_{0}$ is the laser wavelength), where the optical nonlinear effects caused by the strong laser in plasmas become significant, making the manipulation of polarization for high-power lasers challenging. For example, the nonlinear plasma wake (NPW) can be excited in plasmas of the density of 10^18 ^cm^−3^, and various nonlinear effects occur, e.g., laser self-phase modulation, self-steepening, and relativistic self-focusing^19^. These nonlinear effects change the evolution characteristics of drive laser significantly^20–25^, leading to some novel phenomena, e.g., photon deceleration/acceleration and asymmetric pulse compression^26–33^. Especially, the laser photons located at the negative refractive index gradient, i.e., $\partial \eta /\partial \xi < 0$, undergo frequency down-shifting to the mid-infrared (mid-IR) pulse via the photon deceleration^26–31^. Here, $\xi =x-{ct}$ is the laser-rest frame, $c$ is the speed of light in vacuum, and $x$ is the Cartesian coordinate. The achieved relativistic mid-IR pulse plays a crucial role in ultrafast science, strong-field physics, and laboratory astrophysics, such as investigation of electron dynamics^34,35^, terahertz (THz) emission^36^, high-order harmonic generation^37^, particle acceleration^38,39^, etc. In the magnetized plasma, the photon deceleration becomes even more significant since the refractive index is changed by the electron cyclotron gyration. Considering the fact that the FR angle is proportional to the square of the instaneous laser wavelength, i.e., $\Delta \Psi \propto {\lambda }^{2}$, the FR together with the photon deceleration will be affected significantly in the magnetized plasmas. This poses greater challenges for the generation and manipulation of high-power mid-IR pulses, which is a critical question in relativistic plasma magneto-optics yet to be understood and discussed before.

Here, we propose a novel compact rotator for relativistic mid-IR pulses via the interplay of Faraday effect, the relativistic effect, and the photon deceleration in magnetized plasmas, i.e., frequency-variable Faraday rotation (FVFR). The rotator has been achieved by driving the longitudinally magnetized underdense plasma with a relativistic LP laser pulse of several tens TW. A theoretical model has been developed to explore the electron dynamics and underlying physics. The photon deceleration coupling with the relativistic Faraday effect is capable of not only generating long-wavelength laser pulse but also polarizing the generated beams in a compact and efficient manner. It is shown that the predicted FR angle and the laser wavelength agree well with the three-dimensional (3D) particle-in-cell (PIC) simulations. For the first time, we realized the generation and precise polarization manipulation of the relativistic mid-IR pulses in magnetized plasmas. Such a rotator via FVFR is valid from mid to far-infrared and even THz waveband, which is distinct from the conventional methods^40,41^, opening a new avenue in relativistic plasma magneto-optics and making a major step towards the development of future relativistic plasma optical devices. Especially, the FVFR may contribute to the explanation of frequency-dependent polarization evolution in the mid and far-infrared wave^42,43^ and the fast radio bursts (FRBs)^44,45^ in the universe.

## Results

### Concept and physical scheme

**Fig. 1:**
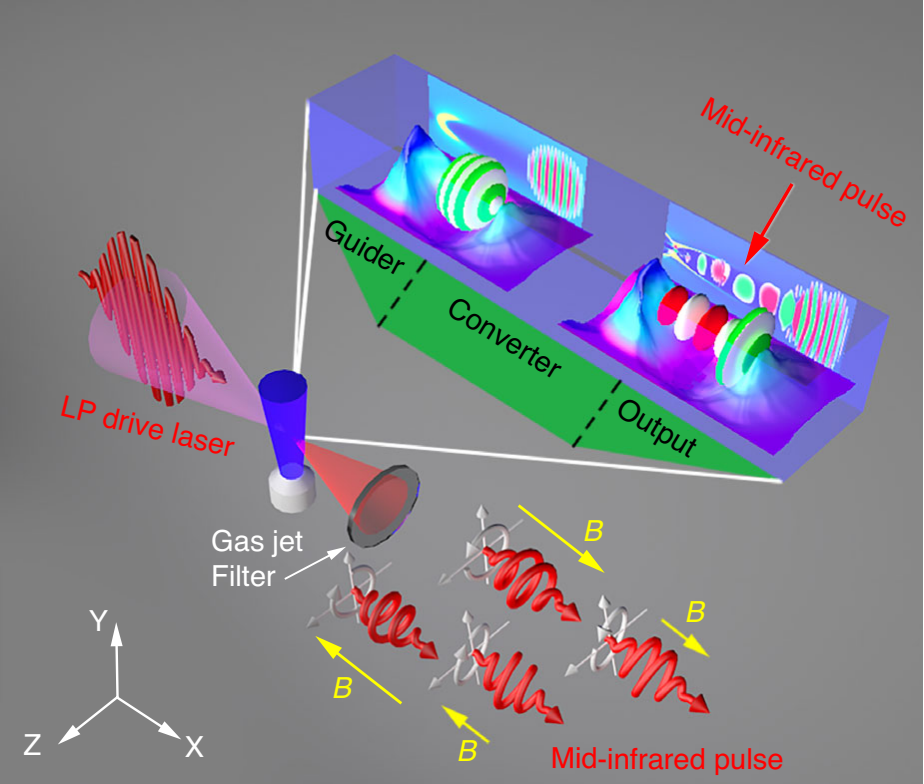
The schematic of magnetized plasma rotator based on the frequency-variable Faraday rotation (FVFR) in the nonlinear plasma wake (NPW). The rotator consists of three modules: guider, converter, and output. The yellow arrows with different lengths indicate the direction and intensity of the external magnetic field. The insets show the process of laser-gas interaction and the evolution of laser pulse in magnetized plasmas

Figure 1 shows the schematic diagram of the magnetized plasma rotator, which consists of three modules, the guider, converter, and output. In the guider corresponding to the density up-ramp, the drive laser propagates in the magnetized plasmas, exciting the NPW with a curving refractive index. Especially, the gradually increasing density can guide the drive laser propagation while suppressing the boundary electron injection. In the converter corresponding to the density plateau, the drive laser is split into a left-handed (LH) and a right-handed (RH) CP subpulse, serving as a core component of the magnetized plasma rotator. The drive laser is transformed into relativistic mid-IR pulses with precisely controlled polarization states via the FVFR. The output corresponding to the density down-ramp, transports the mid-IR pulse out of the plasma efficiently while preserving its beam quality. In the following, we first develop an analytical model to explore the interplay between the Faraday effect, the relativistic effect, and the photon deceleration in the magnetized plasma.

Assuming a constant external magnetic field ${\boldsymbol{B}}=\sigma {B}_{0}{\boldsymbol{x}}$ along the laser axis, where $\sigma =\pm\! 1$ corresponds to along (against) the laser propagation direction and ${B}_{0}$ is the intensity of the magnetic field. The refractive index and phase velocity of the resulting LHCP and RHCP subpulses can be obtained by the wave equation of relativistic CP pulse in the magnetized plasma as demonstrated in Eqs. (S1)–(S5) (see Supplementary Information). The FR angle can be calculated by $\Delta \Psi ={\int }_{{s}_{1}}^{{s}_{2}}{\Delta }_{{\rm{p}}}{kds}/2$ (ref. ^46^), where $s={s}_{2}-{s}_{1}$ is the interaction length, $k$ is the laser wave number, and ${\Delta }_{{\rm{p}}}=\left|{v}_{{\rm{pL}}}-{v}_{{\rm{pR}}}\right|/c$ is the difference of phase velocity of LHCP (${v}_{{\rm{pL}}}$) and RHCP (${v}_{{\rm{pR}}}$) subpulses, which can be written as (see more details in Supplementary Section 1)

1 $${\Delta }_{{\rm{p}}}\approx \frac{{\omega }_{{\rm{p}}}^{2}}{{\omega }^{2}}\frac{n}{{n}_{0}}\frac{\Omega }{1-{\Omega }^{2}}$$

where ${\omega }_{{\rm{p}}}=\sqrt{4\pi {n}_{0}{e}^{2}/{m}_{{\rm{e}}}}$ is the plasma frequency, $\Omega ={\omega }_{{\rm{c}}}/\omega \gamma$, $\omega =2\pi c/\lambda$ is the instaneous laser angular frequency, and ${\omega }_{{\rm{c}}}=e{B}_{0}/{m}_{{\rm{e}}}c$ is the electron cyclotron frequency. $e$, ${m}_{{\rm{e}}}$, $n$, ${n}_{0}$, $\gamma =\sqrt{1-{v}^{2}/{c}^{2}}$, and $v$ are the electron charge, the rest mass, the perturbation electron density, the initial electron density, the electron Lorentz factor, and the local electron velocity, respectively. In the Faraday rotation, the linearly polarized laser pulse can be viewed as a combination of two circularly polarized subpulses with opposite chirality. During the relativistic laser splitting in magnetized plasma, the electrons experience superimposed electric field force and the Lorentz force from both resultant LHCP subpulse and RHCP subpulse. Thus, the electron density $n$ and the electron Lorentz factor $\gamma$ in Eq. (1) refer to the local electron density and Lorentz factor driven by the compound laser pulse. Note that the Faraday rotation angle is integrated over considerable lengths that are much longer than the laser wavelength, so that the contribution of fast oscillation terms of relativistic factor $\gamma$ and density $n$ driven by LP laser can be averaged. Thus, the FR angle in the relativistic regime can be written as

2 $$\Delta \Psi =\sigma \frac{{e}^{3}}{2\pi {m}_{{\rm{e}}}^{2}{c}^{4}}{\int }_{{s}_{1}}^{{s}_{2}}n{B}_{0}\frac{{\lambda }^{2}}{{\gamma }^{2}}\frac{1}{1{-\sigma }^{2}{\Omega }^{2}}{ds}$$

When the magnetic field is small, ${\omega }_{{\rm{c}}}^{2}\ll {\omega }^{2}$, $\Omega \sim 0$, and Eq. (2) can thus be simplified as $\Delta \Psi =(\sigma {e}^{3}/2\pi {m}_{{\rm{e}}}^{2}{c}^{4})({\int }_{{s}_{1}}^{{s}_{2}}{\lambda }^{2}n{B}_{0}/{\gamma }^{2}{ds})$. Within the laser electron interaction region, $\Delta \Psi$ is approximately proportional to ${\lambda }^{2}/{\gamma }_{\max }^{2}$. Obviously, in the non-relativistic regime, i.e., $\gamma =1$, Eq. (2) degenerates to the classical FR, i.e., $\Delta \Psi =(\sigma {e}^{3}/2\pi {m}_{{\rm{e}}}^{2}{c}^{4})({\int }_{{s}_{1}}^{{s}_{2}}{\lambda }^{2}n{B}_{0}{ds})$. In the relativistic regime, the Lorentz factor plays an important role, making the FR of a short wavelength ($\lambda /{\rm{\mu }}{\rm{m}} < {\gamma }_{\max }$) pulse more difficult. Fortunately, the challenge can be overcome by generating a long-wavelength ($\lambda /{\rm{\mu }}{\rm{m}} > {\gamma }_{\max }$) pulse in the magnetized NPW, e.g., mid-IR pulses or THz waves, to mitigate the relativistic effect.

The converter as shown in Fig. 1 serves as such a role, where the laser frequency can be down-shifted from near-infrared to mid and even far-infrared waveband via the photon deceleration^26–31^. Here, the laser wavelength changes in the NPW during a short time $\Delta \tau$ can be estimated by $\lambda -{\lambda }_{0}={\lambda }_{0}\Delta \tau \partial {v}_{{\rm{p}}}/\partial \xi$ (ref. ^21^) with $\Delta \tau \partial {v}_{{\rm{p}}}/\partial \xi$ the difference of the phase velocity between the two adjacent crests. Thus, the instaneous laser wavelength can be expressed as

3 $$\lambda ={\lambda }_{0}\left[1-{\int }_{{s}_{1}}^{{s}_{2}}\left({\eta }^{-2}\frac{\partial \eta }{\partial \xi }\right){ds}\right]$$

Since the plasma in the converter is uniform, we can assume that the drive laser pulse is quasi-static and its envelope varies slowly within the converter due to the fact that the laser envelope characteristic time is much larger than the laser duration^47^ (see Materials and methods)_._ In quasi-static approximation (QSA)^19,47,48^, the refractive index gradient also changes slowly, so that the term $1-{\int }_{{s}_{1}}^{{s}_{2}}{(\eta }^{-2}\partial \eta /\partial \xi ){ds}$ can be approximatively rewritten as $1-{\eta }^{-2}(\partial \eta /\partial \xi )s$. Due to ${\omega }_{{\rm{c}}}^{2}\ll {\omega }^{2}$, the term ${\Omega }^{2}$ in Eq. (2) can be also ignored. Combining Eq. (2) and Eq. (3), the FR angle can be written as

4 $$\Delta \Psi =\sigma \frac{{e}^{3}{\lambda }_{0}^{2}}{2\pi {m}_{{\rm{e}}}^{2}{c}^{4}}{\int }_{{s}_{1}}^{{s}_{2}}n{B}_{0}\frac{1}{{\gamma }^{2}}{\left(1-{\eta }^{-2}\frac{\partial \eta }{\partial \xi }s\right)}^{2}{ds}$$

As compared to the classical case of FR, the FR in the relativistic regime above becomes complicated and different, which is designated as FVFR. In our scheme, the applied external magnetic field is not more than 1000 T, i.e., ${\omega }_{{\rm{c}}}^{2}\le 0.009{\omega }_{0}^{2}$, so the magnetic field has limited effect on the NPW. Therefore, the relativistic fluid equation degenerates to the case without the magnetic field (see Materials and methods), and the equations of electron density and the Lorentz factor are $n={n}_{0}\{1+[(1+{a}_{{\rm{L}}}^{2}/{\left(1+\phi \right)}^{2})]/2\}$ and $\gamma =[1+{a}_{{\rm{L}}}^{2}+{\left(1+\phi \right)}^{2}]/[[2(1+\phi )]$ (ref. ^47^), respectively. Here, ${a}_{{\rm{L}}}$ and $\phi$ are the laser dimensionless vector potential and scalar potential, respectively. $\phi$ can be solved analytically via the QSA^19,47,48^. Finally, Eq. (4) together with Eq. (3) can thus predict the FR angle and the laser wavelength evolution in the relativistic magnetized plasma.

### 3D PIC simulations

To demonstrate the plasma rotator via the FVFR in the NPW, a series of 3D PIC simulations have been performed by using the OSIRIS code^49^. The longitudinally magnetized plasma has a 400 μm linear up-ramp (guider), a 900 μm plateau (converter), and a 400 μm linear down-ramp (output) with the maximum density of ${n}_{0}=4.5\times {10}^{18}$ cm^−3^. The drive laser pulse is characterized by the dimensionless parameter of ${a}_{{\rm{L}}}=3$, a wavelength of 1 μm, a full width of 33 fs, a focal size of 15 μm, and a peak power of about 43 TW, polarized along the $y$ direction.

**Fig. 2:**
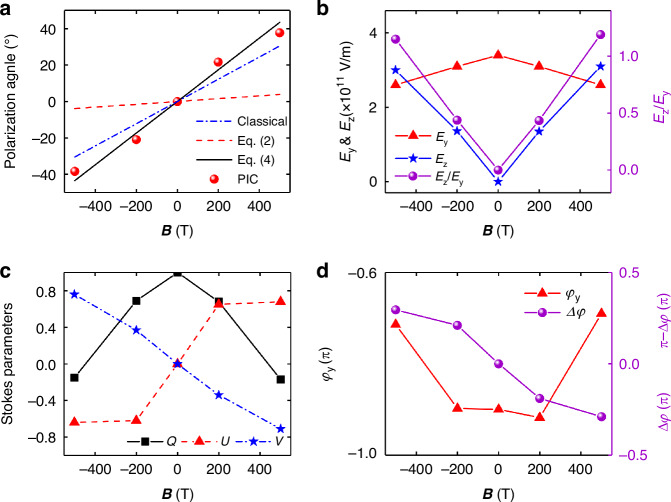
The relationship between mid-IR polarization and external magnetic field. **a** The polarization angle from analytical model and PIC simulations varying with the external magnetic fields. Here, the red points are calculated by $\Delta \Psi =\arctan (U/Q)/2$ with $Q$ and $U$ being the Stokes parameters at the peak position of mid-IR envelope. **b** ${E}_{y}$ and ${E}_{z}$ of mid-IR pulses as well as the intensity ratio (${E}_{z}/{E}_{y}$) as a function of the magnetic field $B$. **c** The Stokes parameters at the peak position of mid-IR envelope at different magnetic fields. **d** The phase change of ${E}_{y}$ (${\varphi }_{y}$) and the phase difference of ${E}_{y}$ and ${E}_{z}$ ($\Delta \varphi$) as a function of the magnetic field $B$

Figure 2 presents the beam characteristics of generated mid-IR pulse at different magnetic field $B$ at $x=1300$ μm. It is shown that the numerical center wavelength of 6.2 μm is comparable to the analytically expected wavelength of ~5.5 μm by Eq. (3). In Fig. 2a, the analytical results of Eq. (2), Eq. (4), and the classical FR equation are compared with the 3D PIC simulations. Here, Eq. (2) is the Faraday rotation angle in the relativistic regime but does not yet consider the wavelength change, so the relativistic Faraday rotation angle becomes about ${\gamma }^{2}$ times smaller than the classical Faraday rotation. Equation (4) is the Faraday rotation in the relativistic regime considering the interplay of Faraday effect, the relativistic effect, and the photon deceleration. It is indicated that the analytical model predicts well the PIC simulations, validating the magnetized plasma rotator in the relativistic regime. Figure 2b shows the electric field components ${E}_{y}$ and ${E}_{z}$ of mid-IR pulses as well as the intensity ratio (${E}_{z}/{E}_{y}$) under different external magnetic fields. It can be seen that, as the magnetic field strength increase, ${E}_{z}$ increases while ${E}_{y}$ decreases. At $B=500$ T, the intensity ratio ${E}_{z}/{E}_{y}$ exceeds 1.0, indicating a polarization rotation angle greater than 45°. In order to precisely describe the mid-IR polarization, Fig. 2c presents the Stokes parameters $Q$, $U$, and $V$ (see Materials and methods) at the peak position of the mid-IR pulse envelope under different magnetic fields, providing a clear visualization of the polarization state of the mid-IR pulse. The results show distinct nonreciprocal behavior in the FVFR, with opposite polarization and chirality observed at $B > 0$ and $B < 0$. Specifically, the mid-IR pulse shows LHCP state when $B > 0$, with its polarization plane rotating towards +45°, i.e., $Q$ goes from 1 to about 0, with $U > 0$ and $V < 0$. Conversely, the polarization plane rotates towards -45° with a RHCP state when $B < 0$, i.e., $U < 0$ and $V > 0$. In addition, the carrier envelope phase (CEP) of the mid-IR pulse is also an important parameter, especially for few-cycle pulses. Figure 2d presents the phase change of ${E}_{y}$ (${\phi }_{y}$) and the phase difference of ${E}_{y}$ and ${E}_{z}$ ($\Delta \phi$) as a function of the magnetic field $B$. Previous studies have demonstrated that in the photon deceleration without an external magnetic field, the CEP of the generated mid-IR pulse remains phase-locked to that of the drive laser^26,28^. However, under an external longitudinal magnetic field, ${\varphi }_{y}$ shows significant $B$-field intensity dependence due to Faraday rotation, which approaches $-0.7$π at $B > 400$ T. This is because the group velocity despersion (GVD) makes the LHCP subpulse of drive laser, which is mainly responsible for generating the mid-IR pulses, slightly divorce from plasmas, resulting in a smaller phase shift. Similarly, the phase difference $\Delta \varphi$ also depends on the magnetic field, and the mid-IR pulse becomes circularly polarized when $\Delta \varphi =0.5$π or ${\rm{\pi }}-\Delta \varphi =0.5$π.

**Fig. 3:**
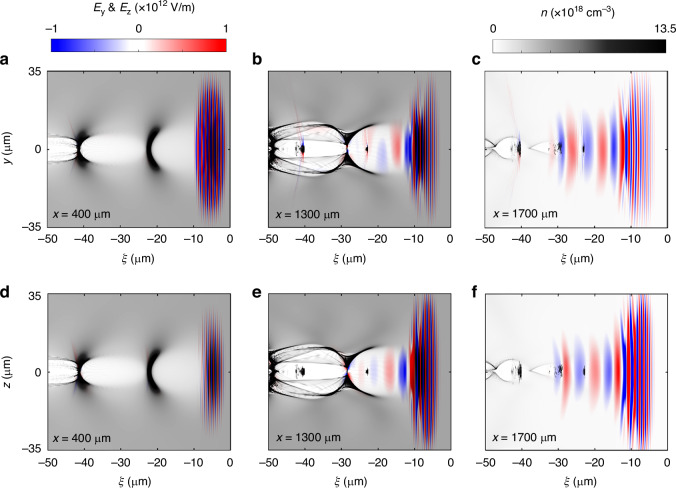
The evolution of transverse electric field and background electron density. The snapshots of transverse electric field ${E}_{y}$ (**a**–**c**), ${E}_{z}$ (**d**–**f**) and the background electron density at $x=400$ μm, 1300 μm, and 1700 μm, respectively

Here, we present an extreme case to exemplify the generation of the CP mid-IR pulse at $B=1000$ T. Under these conditions, the electron cyclotron frequency remains substantially smaller than the drive laser frequency (${\omega }_{{\rm{c}}}^{2}=0.009{\omega }_{0}^{2}$), ensuring limited effect of the external magnetic field on the NPW. Such a strong magnetic field can be already generated by laser-matter interaction^50–57^, e.g., laser solid-target interaction^52^, a laser-driven capacitor-coil target or a snail target^51,56,57^, etc. The magnetic fields of more than 1000 T generated via these techniques can exist in excess of 1 ns (ref. ^51^), significantly exceeding the characteristic interaction durations in our scheme. Figure 3 shows the evolution of the transverse electric field and the electron density at $x=400$ μm, 1300 μm and 1700 μm with $B=1000$ T when the simulation box front reaches (details in Supplementary Figs. S1 and S2 in the Supplementary Information). It can be clearly seen that a stable plasma wake (bubble)^58,59^ can be excited in the magnetized plasma, and the drive laser pulse is located always at the front of the bubble. At $x=400$ μm (i.e., guilder), the ${E}_{z}$ component of the drive laser has appeared due to the relativistic Faraday rotation, as shown in Fig. 3d. In the converter, the long-wavelength mid-IR pulse is effectively generated via the photon deceleration, which later slips backwards into the plasma bubble relative to the drive laser pulse due to the GVD. Confined by the bubble sheath, the spot size of the mid-IR pulse matches the bubble diameter, as shown in Fig. 3b, e. In the output, the mid-IR pulse leaves the plasma while preserving its beam quality as shown in Fig. 3c, f.

**Fig. 4:**
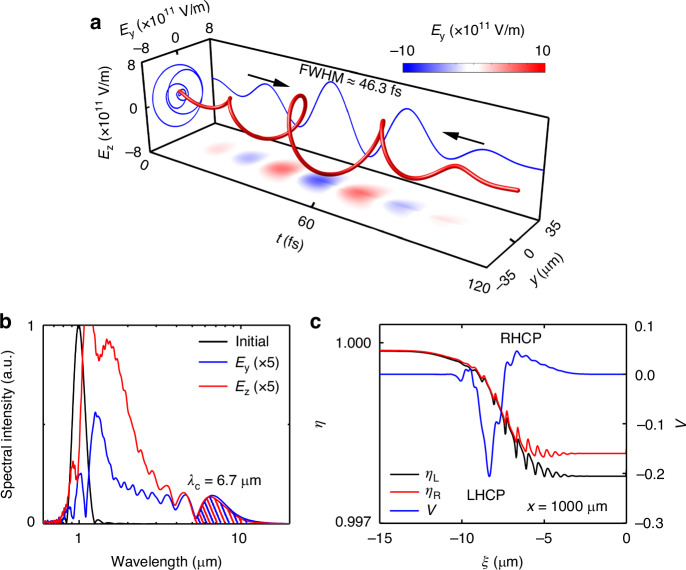
The mid-IR pulse beam quality. **a** The temporal profile of the on-axis mid-IR electric field at $x=1700$ μm. **b** Spectral distribution of on-axis electric field at $x=0$ μm (black) and $x=1700$ μm (red and blue). **c** The distributions of refractive index of LCHP (black), RHCP subpulses (red), and the Stokes parameter $V$ of drive laser (blue) at $x=1000$ μm

Figure 4 exhibits the beam quality of the generated mid-IR pulse in an extreme case with $B=1000$ T. It is shown that a relativistic mid-IR pulse filtered by the bandpass filter can be generated with a full width at half maximum (FWHM) duration of about 46.3 fs, a spot size of 20.8 μm, and a peak electric field ${E}_{{\rm{MIR}}}\approx 6.1\times {10}^{11}$ V m^−1^. The dimensionless laser electric field is ${a}_{{\rm{MIR}}}\approx 1.3$, and the spatial average polarization degree defined by ${P}_{{\rm{s}}}=\sqrt{{\left\langle Q\right\rangle }_{{\rm{s}}}^{2}+{\left\langle U\right\rangle }_{{\rm{s}}}^{2}+{\left\langle V\right\rangle }_{{\rm{s}}}^{2}}/{\left\langle I\right\rangle }_{{\rm{s}}}$ (ref. ^60^) can reach 0.94 (more polarization information of the output pulse are shown in Supplementary Fig. S3 of the Supplementary Information). In addition, after the pulse leaves the plasma, the divergence angle of mid-IR pulse can be estimated as $\theta =\arctan ({r}_{{\rm{s}}}/L)\approx$ 6.4°, where ${r}_{{\rm{s}}}$ is the radius of the spot size at $x=1900$ μm, and $L=200$ μm is the propagation length. Figure 4b presents the spectral distribution of the on-axis electric field at $x=0$ μm and $x=1700$ μm, respectively. One sees that the mid-IR pulse has a spectral width of 5–25 μm and an energy conversion efficiency of 1.8%, centered at ${\lambda }_{{\rm{c}}}=6.7$ μm. As expected in Fig. 4b, ${E}_{y}$ and ${E}_{z}$ of the mid-IR pulse have the same amplitude, justifying the LHCP pulse generation. The refractive index of resulting LHCP and RHCP subpulses, and the Stokes parameter $V$ of the drive laser are also shown in Fig. 4c. Similar to the classical Faraday effect^11–13^, the different refractive index causes different phase velocity and group velocity, leading to the splitting of the LHCP pulse and the RHCP pulse in the magnetized plasma, according to Supplementary Eqs. (S3) and (S4). However, the excitation of plasma bubble with negligible electrons leads to incomplete relativistic laser splitting. Therefore, under an extreme magnetic field of 1000 T, as shown in Fig. 4c, the slower LHCP subpulse slips backward into a region with a smaller negative refractive index gradient, i.e., $\partial {\eta }_{{\rm{L}}}/\partial \xi < \partial {\eta }_{{\rm{R}}}/\partial \xi$, where it becomes the dominant source of the LHCP mid-IR pulse. Though this case corresponds to the magnetic field of *B* = 1000 T, the magnetized plasma rotator is also valid at a much lower magnetic field, e.g., *B* = 500 T (see Supplementary Fig. S4), which can be already generated by laser-driven coil-targets at GEKKO-XII and LULI2000 laser facilities^51,53^. In this case, the achieved mid-IR pulse is also CP with intensity of ${E}_{y}\approx {E}_{z}\approx 1.8\times {10}^{11}$ V m^−1^.

**Fig. 5:**
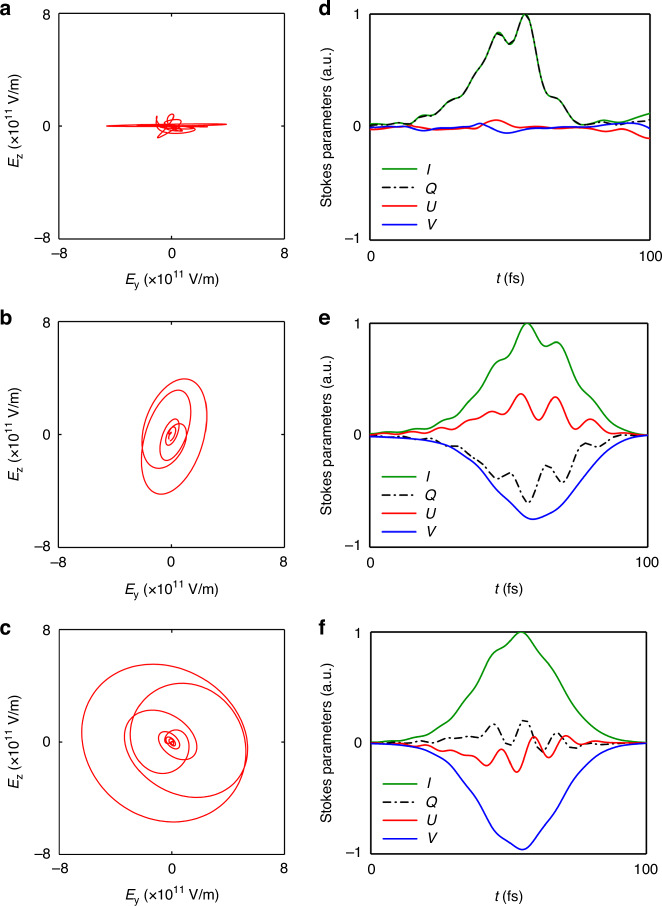
The polarization of mid-IR pulse. The Lissajous figures and the Stokes parameters of the generated mid-IR pulse at the end of output with different magnetic fields of $B=0$ T (**a**, **d**), 500 T (**b**, **e**), and 1000 T (**c**, **f**), respectively

In the converter, the generated mid-IR pulse gradually slips backward into the bubble and moves forward together with the drive laser because of the GVD. After $x=1300$ μm, the drive laser enters into the output. In this stage, the bubble enlarges gradually in the down-ramp, making the center of the bubble retreat relative to the mid-IR pulse at a small velocity. Figure 5 exhibits the Lissajous figures and the Stokes parameters of the mid-IR pulse with different $B$ at the end of the output. One clearly sees that, regardless of the intensity of the magnetic field, the generated mid-IR pulse is left-handed when the external magnetic field is along the laser propagation direction. The polarization of mid-IR pulse is consistent with the drive laser when there is no external magnetic field, i.e., $Q=1$, $U\approx 0$, and $V\approx 0$, as shown in Fig. 5d. With the increase of the magnetic field, the generated mid-IR pulse becomes elliptically polarized, e.g., $Q\approx -0.60$, $U\approx 0.31$, and $V\approx -0.74$ at $B=500$ T as shown in Fig. 5e. In an extreme case, the LHCP mid-IR pulse ($V\approx -0.96$) can be obtained at $B=1000$ T in Fig. 5f. As expected, a RHCP mid-IR pulse can also be generated when the magnetic field revers the direction. By use of a long-wavelength drive laser, e.g., CO_2_ laser with ${\lambda }_{0}=10$ μm, the proposed magnetized plasma rotator, at an appropriate parameter of the external magnetic field, can also be used to modulate the polarization of far-infrared pulse, e.g., THz wave^31^. Compared with the polarization-tunable THz wave emitting in magnetized gas^40,41^, the proposed compact magnetized plasma rotator based on FVFR can manipulate the polarization of relativistic THz waves in a different manner of optical spectrum evolution. Such a relativistic mid and far-IR pulse with tunable polarization have demonstrated a crucial role in strong fields and attosecond science, e.g., the above-threshold ionization^35,61–63^ and charge particle acceleration^38,64–66^, etc.

## Discussion

**Fig. 6:**
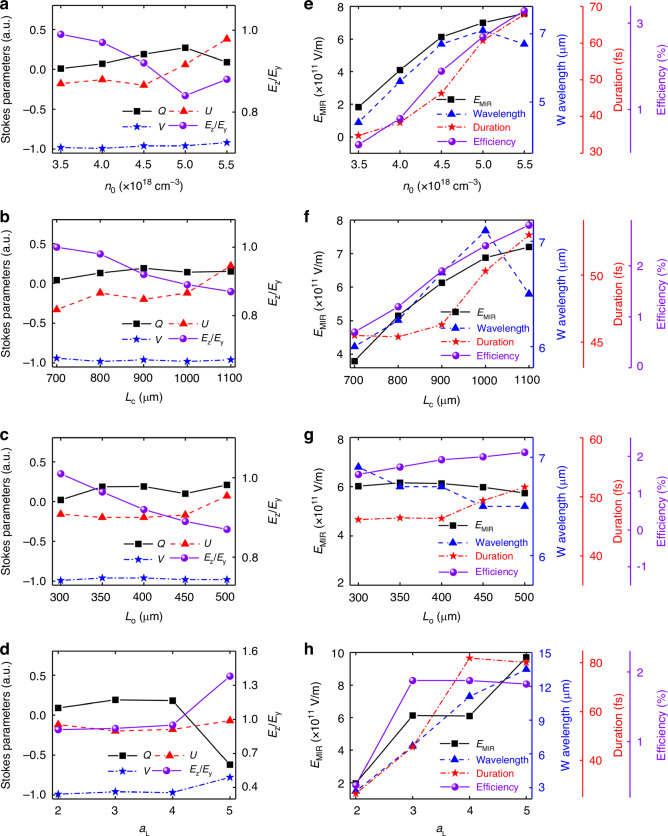
The robustness of the magnetized plasma rotator. **a**–**d** The Stokes parameters, the eccentricity ${E}_{z}/{E}_{y}$, and **e**–**h** the beam parameters and the efficiency of the generated mid-IR pulse with different plasma density ${n}_{0}$, converter length ${L}_{{\rm{c}}}$, output length ${L}_{{\rm{o}}}$, and the initial laser intensity ${a}_{{\rm{L}}}$. In all cases, $B=1000$ T

We now discuss the robustness of the magnetized plasma rotator in terms of the plasma parameters and laser intensity. As shown in Fig. 6a–d, the Stokes parameters $Q$ and $U$ change with the electron density ${n}_{0}$, the length of converter ${L}_{{\rm{c}}}$ and output ${L}_{{\rm{o}}}$, and the initial laser dimensionless parameter ${a}_{{\rm{L}}}$, but $V$ is always ~−1 with ${E}_{z}/{E}_{y}$ larger than 0.84 (except the case of ${a}_{{\rm{L}}}=5$ with ${E}_{z}/{E}_{y}=1.4$), indicating the stable generation of the LHCP mid-IR pulse. On the other hand, one can see in Fig. 6e that the intensity ${E}_{{\rm{MIR}}}$ and the center wavelength of the mid-IR pulse, together with the laser energy conversion efficiency and the duration of mid-IR pulse, can be improved by increasing the plasma density. This is because a larger ${n}_{0}$ provides a smaller refractive index gradient to expedite the frequency down-shifting. However, at ${n}_{0}=5.5\times {10}^{18}$ cm^−3^, the photon acceleration occurred at the tail of the plasma bubble, leading to a shortened wavelength. Figure 6f also shows that the field strength ${E}_{{\rm{MIR}}}$, the duration, and the energy conversion efficiency are improved by using a longer ${L}_{{\rm{c}}}$, since a longer ${L}_{{\rm{c}}}$ enables more photons to convert to the mid-IR pulse. Similarly, the photon acceleration shortens the mid-IR pulse wavelength when ${L}_{{\rm{c}}}$ exceeds the optimal conversion length. The longer ${L}_{{\rm{o}}}$ makes the central wavelength short and the efficiency higher because the mid-IR pulse undergoes the photon acceleration when it is located at the tail of the bubble, as shown in Fig. 6g. However, the field strength ${E}_{{\rm{MIR}}}$ decreases slightly because the longer ${L}_{{\rm{o}}}$ leads to a larger bubble, making the duration of the mid-IR pulse longer. This indicates that the proposed magnetic plasma rotator is highly robust and valid for a wide plasma parameter range of ${n}_{0}$, ${L}_{{\rm{c}}}$, and ${L}_{{\rm{o}}}$. In addition, Fig. 6h shows that as the initial laser intensity increases, the center wavelength of the mid-IR pulse lengthens while the intensity ${E}_{{\rm{MIR}}}$, the laser energy conversion efficiency and the duration of mid-IR pulse initially increase before the saturation. It should be noted that, at ${a}_{{\rm{L}}}=1$, the mid-IR pulse generation within the plasma bubble is suppressed (not shown here). However, when ${a}_{{\rm{L}}}=5$, the generated mid-IR pulse wavelength reaches ~13.5 μm. This induces a significant polarization rotation, yielding an elliptically polarized mid-IR pulse.

To further illustrate the correctness of the simulation results and the operability of the proposed rotator, we discuss additional potential issues, e.g., the spatially non-uniform external magnetic field obtained by the laser driven coil-target, the directed motion of the gas jet, and the plasma temperature. In practice, the external magnetic field, taking the laser driven coil-target for example, conforms to the Biot-Savart Law. In this case, the CP mid-IR pulse can still be generated (see Supplementary Fig. S5 in the Supplementary Information). In our scenario, the plasma is produced by a gas jet, which can operate at subsonic or supersonic speeds, as schematically shown in Fig. 1. Since the movement distance of the plasma ejection during the interaction time is much less than 1 μm, the directed motion of the plasma ejection does not affect the FVFR and the generation of the mid-IR pulse (see Supplementary Fig. S6). Similarly, our additional simulations show that the plasma temperature effects can be ignored, as demonstrated in Supplementary Fig. S7, where we consider two cases of different initial plasma temperature, $T=10$ eV and 50 eV, respectively. Finally, the output mid-IR pulse can be filtered by a well-designed IR beam splitter coated with indium tin oxide in experiments^27^, and the filtered mid-IR pulses can be used in exploring electron dynamics, e.g., the strong-field ionization^35^.

In summary, we have proposed a novel compact magnetized plasma rotator based on the interplay of Faraday effect, the relativistic effect, and the photon deceleration, i.e., the FVFR, in magnetized NPW. With the rotator, the laser frequency can be down-shifted to the mid-IR band via photon deceleration, and the polarization of the generated mid-IR pulse can be controlled efficiently by the external magnetic fields. Full 3D PIC simulations indicate that the pulse wavelength and rotation angle agree well with the developed analytical model, providing a basis for designing rotators in experiments. It is the first time to directly modulate and manipulate the polarization of relativistic mid-IR pulses in magnetized plasmas. Such a novel plasma rotator via the FVFR is valid from mid to far-infrared and even THz waveband, offering new opportunities for strong-field physics, attosecond science, and laboratory astrophysics. Especially, this shall open a new avenue in relativistic plasma magneto-optics, e.g., the generation of tens of terawatt polarization-tunable mid-IR pulses on PW laser facilities, making a major step towards the development of future relativistic plasma optical devices. Meanwhile, the observed FVFR may be also highly relevant to some astrophysical phenomena, such as the frequency-dependent polarization evolution in the mid-IR wave and the fast radio bursts (FRBs) in the universe.

## Materials and methods

### The 1D theory of the nonlinear plasma wave generation with external magnetic field

When a relativistic LP laser pulse interacts with a longitudinally strongly magnetized underdense plasma, a nonlinear wake can be excited^19^. This can be described by the one-dimensional (1D) relativistic fluid equations of cold plasmas with stationary ions. Assuming the polarization of the initial laser pulse alone the $y$-direction, and the laser propagation along the $x$-direction, and ignoring the plasma diamagnetism caused by electron rotation, the relativistic fluid equations in the laser-rest frame ($\xi$, $\tau$) can be given by

5 $$\frac{{\partial }^{2}\phi }{\partial {\xi }^{2}}={k}_{{\rm{p}}}^{2}\left(\frac{n}{{n}_{0}}-1\right)$$

6 $$\frac{\partial \left[n\left(1-{\beta }_{x}\right)\right]}{\partial \xi }=\frac{1}{c}\frac{\partial n}{\partial \tau }$$

7 $$\frac{\partial \left(\gamma {\beta }_{x}+\phi -\gamma \right)}{\partial \xi }=\frac{1}{1+{\beta }_{x}}\left[\frac{1}{c}\frac{\partial \left(\gamma {\beta }_{x}\right)}{\partial \tau }+\frac{1}{2\gamma c\left(1-{\sigma }^{2}{\Omega }^{2}\right)}\frac{\partial {a}_{{\rm{L}}}^{2}}{\partial \tau }-\frac{1}{c}\frac{\partial \gamma }{\partial \tau }\right]$$

where ${k}_{{\rm{p}}}$ is the wave number of plasmas, ${\beta }_{x}$ is the longitudinal normalized electron velocity.

Since the plasma in the converter is uniform, we can assume that the drive laser pulse is quasi-static and its envelope varies slowly within the converter due to the fact that the laser envelope characteristic time ${\tau }_{{\rm{e}}} \sim 2{\gamma }_{\max }|{n}_{0}/{n}_{\max }|({\omega }_{0}/{\omega }_{{\rm{p}}})/{\omega }_{{\rm{p}}}\approx 0.4$ ps is much larger than the laser duration ${\tau }_{{\rm{d}}}$^47^, where the density perturbation ${n}_{\max }$ and the relativistic factor ${\gamma }_{\max }$ are obtained at the peak position of the drive laser envelope in PIC simulations. In QSA^19,47,48^, Eqs. (6) and (7) can be integrated to $n\left(1-{\beta }_{x}\right)={n}_{0}$ and $\gamma \left(1-{\beta }_{x}\right)-\phi =1$, respectively. Therefore, Eq. (5) can be rewritten as

8 $$\frac{{\partial }^{2}\phi }{\partial {\left({k}_{{\rm{p}}}\xi \right)}^{2}}=\left(\frac{\gamma }{1+\phi }-1\right)$$

Considering $\gamma (1-{\beta }_{x})-\phi =1$ and $\gamma ={(1+{\gamma }^{2}{\beta }_{x}^{2}+{\gamma }^{2}{\beta }_{y}^{2}+\gamma {\beta }_{z}^{2})}^{1/2}$, $\gamma$ in the QSA can be solved from

9 $$1-2\gamma \left(1+\phi \right)+{\left(1+\phi \right)}^{2}+\frac{{a}_{{\rm{L}}}^{2}}{{\left(1-{\sigma }^{2}{\Omega }^{2}\right)}^{2}}+\frac{{a}_{{\rm{L}}}^{2}{\sigma }^{2}{\Omega }^{2}}{{\left(1-{\sigma }^{2}{\Omega }^{2}\right)}^{2}}=0$$

where ${\beta }_{y}$ and ${\beta }_{z}$ are the transverse normalized electron velocity, which can be solved from the electron momentum equation with an external magnetic field. $\gamma$ and the election density $n=\gamma {n}_{0}/(1+\phi )$ can be put into Eq. (4) to obtain the FVFR angle.

### Particle-in-cell simulations

The initial LP laser electric field with a polarization of $y$ direction can be expressed as follows

10 $$a={a}_{{\rm{L}}}\exp \left(-\frac{{r}^{2}}{{w}_{0}^{2}}\right){\sin }^{2}\left(\frac{\pi t}{{\tau }_{{\rm{d}}}}\right)\sin \left({\omega }_{0}t\right)$$

Here, ${a}_{{\rm{L}}}=3$, ${\lambda }_{0}=1$ μm, ${w}_{0}=15$ μm, and ${\tau }_{{\rm{d}}}=33$ fs. These correspond to the laser peak intensity of ${I}_{0}\approx 1.2\times {10}^{19}$ W cm^−2^, peak power of 43 TW, and pulse energy of about 710 mJ. The initial laser pulse is assumed to arrive at the vacuum-plasma interface at $x=0$ μm.

The 3D PIC simulations were carried out using the code OSIRIS^43^ in Cartesian coordinates. A moving window that is employed along the $x$ direction at the speed of light in vacuum. The simulation box has a size of 50 µm ($x$) $\times$ 70 µm ($y$) $\times$ 70 µm ($z$) with grid cells of 1500 $\times$ 350 $\times$ 350, which corresponds to 30 grid cells per drive laser wavelength in the $x$ direction, sufficient to resolve the physical process of photon deceleration. The number of macroparticles per cell is 2, and the initial particle is stationary. The absorbing boundary condition is used for the electric field and the particle. For the verification of simulation results, we performed additional simulations, and the results show that the high-speed particle collision is not particularly significant in our simulations because the particle collision time is typically much greater than the laser pulse length (see Supplementary Fig. S8). Moreover, the transverse spatial resolution and the macroparticles per cell can not severely affect the FVFR and the mid-IR generation (see Supplementary Figs. S9 and S10).

### Stokes parameters

To describe the polarization state of electromagnetic wave, the Stokes parameters $I$, $Q$, $U$, and $V$ are introduced^11,60,67^. Here, $I={\left|{E}_{y}\right|}^{2}+{\left|{E}_{z}\right|}^{2}$ denotes the intensity distribution of laser pulse. $Q={\left|{E}_{y}\right|}^{2}-{\left|{E}_{z}\right|}^{2}$ denotes the linear polarization component along the $y$ ($Q > 0$) or $z$ ($Q < 0$) direction. $U=2{\rm Re}\{{E}_{y}^{* }{E}_{z}\}$ denotes the linear polarization at +45° ($U > 0$) or -45° ($U < 0$) from the $y$ direction. $V=2{\rm Im}\{{E}_{y}^{* }{E}_{z}\}$ denotes the right-handed ($V > 0$) or left-handed ($V < 0$) circular polarization. Here, ${E}_{y}$ and ${E}_{z}$ are the complex amplitude of the electric field, which can be obtained in PIC simulations, and ${E}_{y}^{* }$ is the complex conjugate of ${E}_{y}$.

## Supplementary information

Supplementary Information for Magnetized plasma rotator for relativistic mid-infrared pulses via frequency-variable Faraday rotation

## Data Availability

The data that support the findings of this study are available from the corresponding author on reasonable request.
