# Supplementary material for: Magnetized plasma rotator for relativistic mid-infrared pulses via frequency-variable Faraday rotation
Source: Light Sci Appl. 2026 Jan 2;15:25. doi: 10.1038/s41377-025-02047-x (PMC12757596; doi:10.1038/s41377-025-02047-x)
Supplement: Supplementary file 1 — Supplementary Information for Magnetized plasma rotator for relativistic mid-infrared pulses via frequency-variable Faraday rotation [file 41377_2025_2047_MOESM1_ESM.pdf]

Supplementary Information for Magnetized plasma rotator for relativistic mid-infrared pulses via frequency-variable Faraday rotation

Dong-Ao Li[1], Guo-Bo Zhang[1], Francesco Pegoraro[2,3], Qian Zhao[4], Wen-Jun Liu[1], Xing-Long Zhu[5], De-Bin Zou[1], Jian-Xing Li[4], Alexander Pukhov[6], Zheng-Ming Sheng[7,8,9], and Tong-Pu Yu[1,*]

[1]College of Science, National University of Defense Technology, Changsha 410073, China.
[2]Physics Department, University of Pisa, Pisa 56127, Italy.
[3]CNR, Istituto Nazionale di Ottica (INO), Pisa 56127, Italy.
[4]Ministry of Education Key Laboratory for Nonequilibrium Synthesis and Modulation of Condensed Matter, Shaanxi Province Key Laboratory of Quantum Information and Quantum Optoelectronic Devices, School of Physics, Xi'an Jiaotong University, Xi'an 710049, China.
[5]Institute for Fusion Theory and Simulation, School of Physics, Zhejiang University, Hangzhou 310058, China.
[6]Institut für Theoretische Physik I, Heinrich-Heine-Universität Düsseldorf, Düsseldorf 40225, Germany.
[7]Key Laboratory for Laser Plasmas (MOE), School of Physics and Astronomy, Shanghai Jiao Tong University, Shanghai 200240, China.
[8]Collaborative Innovation Center of IFSA, Shanghai Jiao Tong University, Shanghai 200240, China.
[9]Tsung-Dao Lee Institute, Shanghai Jiao Tong University, Shanghai, 201210, China.
[*]Correspondence: Tong-Pu Yu (tongpu@nudt.edu.cn)
These authors contributed equally: Dong-Ao Li, Guo-Bo Zhang.

Abstract

The supplementary information is organized as follows. Section 1 presents the detailed derivations of the frequency-variable Faraday rotation theory. Section 2 presents the detailed evolutions of the transverse electric field and the nonlinear plasma wake. Section 3 shows the polarization of the output laser beam. Section 4 gives an example of the plasma rotator with a tailored plasma density and lower external magnetic field. Moreover, some potential issues in simulations and experiments are discussed in Section 5.

1 The frequency-variable Faraday rotation

Since a linearly polarized (LP) laser pulse can be decomposed into a left-handed circularly polarized (LHCP) subpulse and a right-handed circularly polarized (RHCP) subpulse with equal-amplitude, the Faraday rotation (FR) arises from the different phase velocities of these subpulses in a magnetized medium experiencing a longitudinal external magnetic field. The phase velocities of LHCP and RHCP subpulses can be obtained by the wave equation of the relativistic circularly polarized pulse in the magnetized plasma. Assuming a constant external magnetic field $\boldsymbol{B} = \sigma B_0 \boldsymbol{x}$ along the laser axis ($x/-x$ direction), where $\sigma = \pm 1$ corresponds to along (against) the laser propagation direction and B_0 is the intensity of the magnetic field, the wave equation of CP pulse is as follows [1]:

$$\left(\frac{\partial^2}{\partial x^2} - \frac{\partial^2}{c^2 \partial t^2}\right) \boldsymbol{a} = \frac{\omega_{\mathrm{p}}^2}{c^2} \frac{n}{n_0} \frac{\omega}{\omega\gamma - \delta\sigma\omega_{\mathrm{c}}} \boldsymbol{a} \tag{S1}$$

Here, $\boldsymbol{a} = \lambda \boldsymbol{E} e / 2\pi m_{\mathrm{e}} c^2$ is the dimensionless laser electric field with λ the instaneous wavelength of laser pulse and $\boldsymbol{E}$ the electric field intensity of laser pulse. e, m_{e}, n, n_0, γ, c are the electron charge, the rest mass, the perturbation electron density, the initial electron density, the electron Lorentz factor, and the speed of light in vacuum, respectively. $\omega_{\mathrm{p}} = \sqrt{4\pi n_0 e^2 / m_{\mathrm{e}}}$ is the frequency of plasma waves, $\omega = 2\pi c/\lambda$ is the laser angular frequency, and $\omega_{\mathrm{c}} = e B_0 / m_{\mathrm{e}} c$ is the electron cyclotron frequency. $\delta = +1$ and $\delta = -1$ represent the LHCP wave and the RHCP wave, respectively. The electrons manifest fundamentally different trajectories and thus distinct γ factors and density n when driven by linearly polarized laser pulses (with oscillating field directions) and circularly polarized pulses (CP). This dichotomy becomes essential during the relativistic laser pulse splitting in magnetized plasma, where electrons experience superimposed forces from both resultant LHCP subpulse and RHCP subpulse. Thus, the electron density n and the electron Lorentz factor γ in Eq. (S1) refer to the local electron density and Lorentz factor driven by the compound laser pulse.

Form the Eq. (S1), the dispersion relation of CP subpulse can be written as

$$\frac{c^2 k^2}{\omega^2} \simeq 1 - \frac{\omega_{\mathrm{p}}^2}{\omega^2} \frac{n}{n_0 \gamma} \frac{1}{1 - \delta\sigma \frac{\omega_{\mathrm{c}}}{\omega\gamma}} \tag{S2}$$

Therefore, the refractive index for LHCP and RHCP subpulses are

$$\eta_{\mathrm{L,R}} \simeq \left(1 - \frac{\omega_{\mathrm{p}}^2}{\omega^2} \frac{n}{n_0 \gamma} \frac{1}{1 - \delta\sigma \frac{\omega_{\mathrm{c}}}{\omega\gamma}}\right)^{1/2} \simeq 1 - \frac{\omega_{\mathrm{p}}^2}{2\omega^2} \frac{n}{n_0 \gamma} \frac{1}{1 - \delta\sigma \frac{\omega_{\mathrm{c}}}{\omega\gamma}} \tag{S3}$$

the group velocities ($v_{\mathrm{g}} = d\omega/dk$) of LHCP and RHCP subpulses are

$$v_{\mathrm{gL,R}} \simeq c \left(1 - \frac{\omega_{\mathrm{p}}^2}{\omega^2} \frac{n}{n_0 \gamma} \frac{1}{1 - \delta\sigma \frac{\omega_{\mathrm{c}}}{\omega\gamma}}\right)^{1/2} \left(1 + \delta\sigma \frac{1}{2} \frac{\omega_{rmc}}{\omega\gamma} \frac{\omega_{\mathrm{p}}^2}{\omega^2} \frac{n}{n_0 \gamma} \frac{1}{1 - \delta\sigma \frac{\omega_{\mathrm{c}}}{\omega\gamma}}\right)^{-1} \tag{S4}$$

and the phase velocities ($v_{\mathrm{p}} = c\eta^{-1}$) of LHCP and RHCP subpulses are

$$v_{\mathrm{pL,R}} \simeq c \left(1 + \frac{\omega_{\mathrm{p}}^2}{2\omega^2} \frac{n}{n_0 \gamma} \frac{1}{1 - \delta\sigma \frac{\omega_{\mathrm{c}}}{\omega\gamma}}\right) \tag{S5}$$

It can be seen from Eqs. (S3) and (S4) that the refractive index and the group velocities of the LHCP pulse and the RHCP pulse remain divergent. When the external magnetic field is along the laser propagation direction, the group velocity relationship $v_{\mathrm{gL}} < v_{\mathrm{gR}}$ causes the splitting of the subpulses. Moreover, the splitting of LHCP and RHCP subpulses behaves differently in the relativistic region compared with the classical region, due to the excitation of plasma bubble with very few electrons there. When the LCHP subpulse slips backwards into the bubble, the group velocity v_{gL} can approach c, leading to $v_{\mathrm{gL}} > v_{\mathrm{gR}}$. Therefore, the LHCP and RHCP subpulses can incompletely split in this case, which is different from the observations in the extreme Faraday rotation case [2,3].

The FR angle can be calculated by $\Delta\Psi = \int_{s_1}^{s_2} \Delta_{\mathrm{p}} k ds/2$ (Ref. 4), where $s = s_2 - s_1$ is the interaction length, k is the wave number of laser, and $\Delta_{\mathrm{p}} = |v_{\mathrm{pR}} - v_{\mathrm{pL}}|/c$ is the difference of phase velocities of LHCP (v_{pL}) and RHCP (v_{pR}) subwaves. According to the Eq. (S5), Δ_{p} can be rewritten as

$$\Delta_{\mathrm{p}} \approx \frac{\omega_{\mathrm{p}}^2}{\omega^2} \frac{n}{n_0 \gamma} \frac{\frac{\omega_{\mathrm{c}}}{\omega\gamma}}{1 - \frac{\omega_{\mathrm{c}}^2}{\omega^2\gamma^2}} \tag{S6}$$

Note the Faraday rotation angle is integrated over considerable lengths that are much longer than the laser wavelength, so that the contribution of fast oscillation terms of relativistic factor γ and density n can be averaged. Thus, the FR angle of the relativistic LP laser propagating in the magnetized plasma can be written as

$$\Delta\Psi = \sigma \frac{e^3}{2\pi m_{\mathrm{e}}^2 c^4} \int_{s_1}^{s_2} n B_0 \frac{\lambda^2}{\gamma^2} \frac{1}{1 - \sigma^2 \frac{\omega_{\mathrm{c}}^2}{\omega^2\gamma^2}} ds \tag{S7}$$

The Eq. (S7) can degenerate to the classic FR equation without considering the ponderomotive force effect and relativistic effect at a small external magnetic field, i.e., $\Delta\Psi = (\sigma e^3)/(2\pi m_{\mathrm{e}}^2 c^4) \int_{s_1}^{s_2} \lambda^2 n_0 B_0 ds$.

In the bubble regime, the laser frequency can be down-shifted to mid-infrared (mid-IR) waveband via the photon deceleration [5–7,11,12]. The instaneous laser wavelength changing in a short time duration $\Delta\tau$ can be estimated by $\lambda - \lambda_0 = \lambda_0 \Delta\tau \partial v_{\mathrm{p}}/\partial\xi$ [10], where λ_0 is the initial laser wavelength. Thus, the pulse wavelength λ can be expressed as

$$\lambda = \lambda_0 \left[1 - \int_{s_1}^{s_2} \left(\eta^{-2} \frac{\partial\eta}{\partial\xi}\right) ds\right] \tag{S8}$$

By considering the change of laser wavelength, and substituting Eq. (S8) into Eq. (S7), the frequency-variable Faraday rotation (FVFR) angle can be written as

$$\Delta\Psi = \sigma \frac{e^3}{2\pi m_{\mathrm{e}}^2 c^4} \int_{s_1}^{s_2} n B_0 \frac{\lambda_0^2}{\gamma^2} \frac{1}{1 - \sigma^2 \frac{\omega_{\mathrm{c}}^2}{\omega^2\gamma^2}} \left[1 - \int_{s_1}^{s_2} \eta^{-2} \frac{\partial\eta}{\partial\xi} ds\right]^2 ds \tag{S9}$$

Since the plasma in the converter is uniform, we can assume that the drive laser pulse is quasi-static and its envelope varies slowly within the converter due to the fact that the laser envelope characteristic time $\tau_{\mathrm{e}} \sim 2\gamma|n_0/n|(\omega_0/\omega_{\mathrm{p}})/\omega_{\mathrm{p}} \approx 0.4$ ps is much larger than the laser duration [9], where $\omega_0 = 2\pi c/\lambda_0$. In quasi-static approximation [8,9,13], the refractive index gradient also changes slowly, so that the term $[1 - \int_{s_1}^{s_2} (\eta^{-2}\partial\eta/\partial\xi) ds]^2$ can be approximatively rewritten as $[1 - \eta^{-2}(\partial\eta/\partial\xi)s]^2$. When the magnetic field is small, i.e., $\omega_{\mathrm{c}}^2 \ll \omega^2$, the term of $\omega_{\mathrm{c}}^2/\omega^2\gamma^2$ in Eq. (S9) can be ignored. Finally, we can obtain the FVFR

equation in a uniform plasma as follows:

$$\Delta\Psi = \sigma \frac{e^3\lambda_0^2}{2\pi m_e^2 c^4} \int_{s_1}^{s_2} nB_0 \frac{1}{\gamma^2} \left(1 - \eta^{-2}\frac{\partial \eta}{\partial \xi} s\right)^2 ds \tag{S10}$$

2 The evolutions of electric field and plasma wake

The evolutions of the electric field and plasma wake are significant for revealing the underlying physics. Figures S1 and S2 show the snapshots of the transverse electric field E_y, E_z, and the electron density n at the different regions of the density profile. At the stage of guider, the drive laser pulse is guided into the plasma, and the plasma bubbles are excited, as shown in Figs. S1a and S1b. Meanwhile, E_z appears gradually via the Faraday rotation, as shown in Figs. S2a and S2b. At the stage of converter, the drive laser pulse is compressed, and the intensity of drive laser increases via the relativistic self-focusing effect, as shown in Figs. S1c-S1d and Figs. S2c-S2d. During this period, the laser pulse undergoes photon deceleration so that the mid-IR pulse is generated. Due to the group velocity dispersion, the long-wavelength mid-IR pulse slips backwards into the bubble and moves forward together with the drive laser pulse. Meanwhile, a number of electrons can be injected into the plasma bubble, and these electrons does not severely affect the quality of the mid-IR pulse because the interaction distance between the mid-IR pulse and the electrons is relatively short. At the stage of output, the bubble gradually expands since the size of plasma bubble is related to the electron density, i.e. $\lambda_b \propto 1/n_0$. The mid-IR pulse can experience further FVFR as it resides in the front of the bubble again due to the retreat of bubble center.

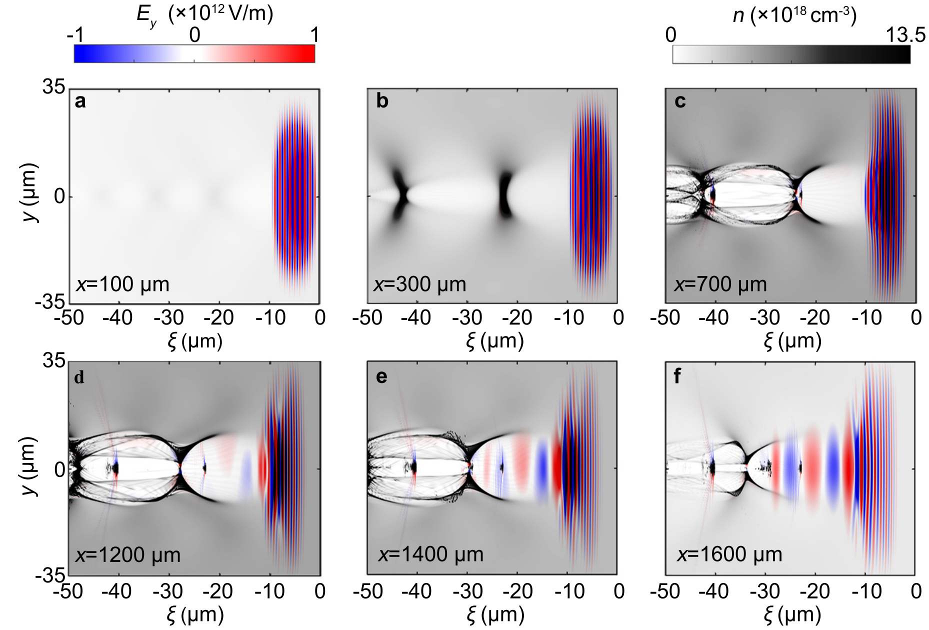

Fig. S1. The evolution of transverse electric field E_y and background electron density. The snapshots of transverse electric field E_y and the electron density n at x = 100 μm (**a**), 300 μm (**b**), 700 μm (**c**), 1200 μm (**d**), 1400 μm (**e**), and 1600 μm (**f**).

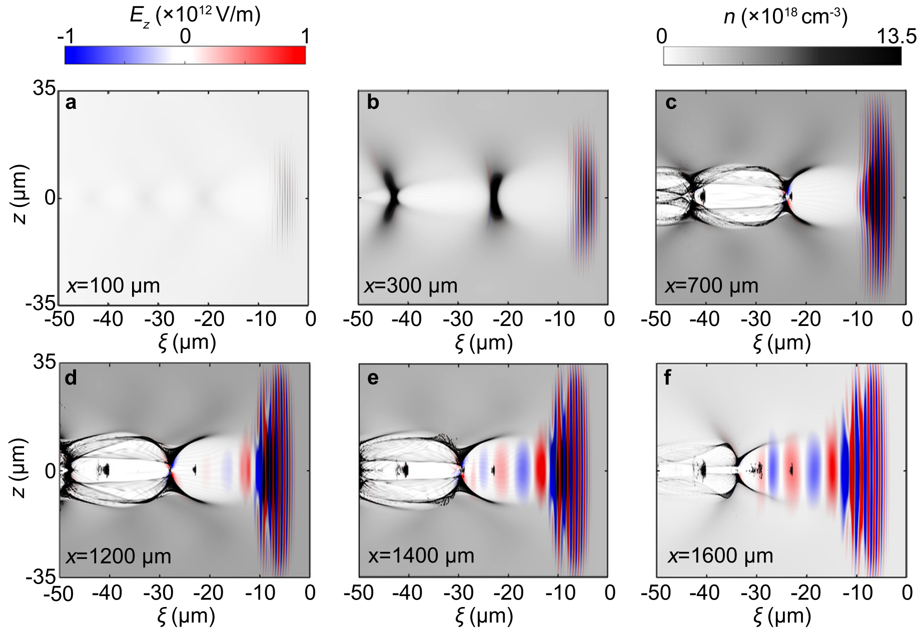

Fig. S2. The evolution of transverse electric field E_z and background electron density. The snapshots of transverse electric field E_z and the electron density n at x = 100 μm (**a**), 300 μm (**b**), 700 μm (**c**), 1200 μm (**d**), 1400 μm (**e**), and 1600 μm (**f**).

3 The polarization of the output laser beam

Figure S3 shows the Lissajous figures and the Stokes parameters for the wavebands of 1.1-2.3 μm, 2.3-5 μm, and 5-25 μm at x = 1700 μm. We see that, the polarization of the output laser beam is different for different wavebands. The electric field of drive laser has a polarization shift from y-direction to z-direction due to the relativistic Faraday effect, as shown in Figs. S3a and S3b. The front part of the drive laser pulse has a right-handed elliptical polarization, while the rear part of the drive laser pulse has a left-handed elliptical polarization. Since a portion of the left-handed drive laser are converted to the left-handed mid-IR pulse, the intensity of the rear part of the drive laser pulse is lower than that of the front part. Figures S3c and S3d show that the short-wavelength mid-IR pulse is left-handed elliptically polarized, and the intensity of the short-wavelength mid-IR pulse is weaker than the drive pulse. The Stokes parameters show that there are multiple polarization states in this waveband due to $Q < 0$ and $U < 0$. This is because the wavelengths in this waveband are close to γ_{max}, which make the different polarization state become clear. On the contrary, the long-wavelength mid-IR pulse approaches circular polarization, as shown in Figs. S3e and S3f, and the intensity of the long-wavelength mid-IR pulse is weaker than the short-wavelength mid-IR pulse. As the Stokes parameters show, in this waveband, most wavelengths become circularly polarized.

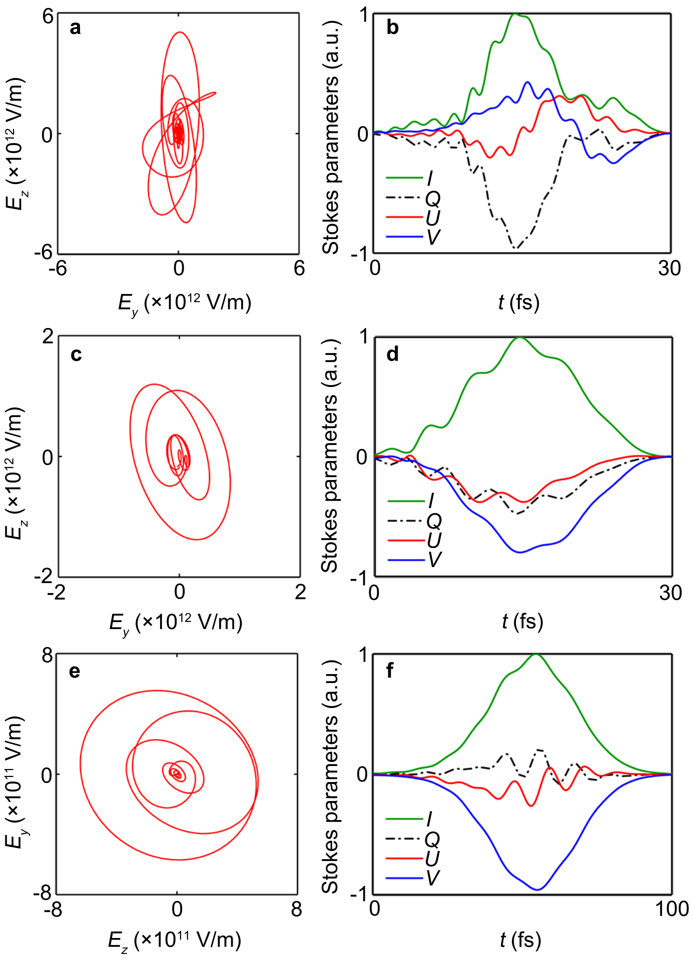

Fig. S3. The polarization of different wavebands. The Lissajous figures and the Stokes parameters for the wavebands of 1.1-2.3 µm (**a**, **b**), 2.3-5 µm (**c**, **d**), and 5-25 µm (**e**, **f**).

4 The plasma rotator with a tailored plasma density structure

Here, we show an example of the plasma polarization rotator at much lower intensity of the external magnetic field B. It is shown that nearly CP mid-IR wave can be generated via a tailored plasma density profile with $B = 500$ T.

In this 3D PIC simulation, the plasma has a tailored density structure with a 400 µm linear up-ramp, a 800 µm plateau of density n_1, a 100 µm linear down-ramp, and a plateau of density n_2, where $n_1 = 4.5 \times 10^{18}$ cm^{-3} and $n_2 = 1 \times 10^{18}$ cm^{-3}, as shown in Fig. S4a. The plasma density structure can be produced by a supersonic round nozzle with a blade covering a portion of the gas jet [6].

At the higher density plateau, the drive laser pulse can be compressed, rotated, and frequency down-shifted. Then the drive laser enters into the lower density plateau, where it can not convert to mid-IR pulse rapidly. The drive laser pulse can further experience the FVFR process, generating a LHCP mid-IR pulse with the wavelength of 6.5 µm. The Lissajous figures and the Stokes parameters of the on-axis mid-IR pulse at $x = 1900$ µm are shown in Figs. S4b and S4c. It can be clearly seen in Fig. S4b that the

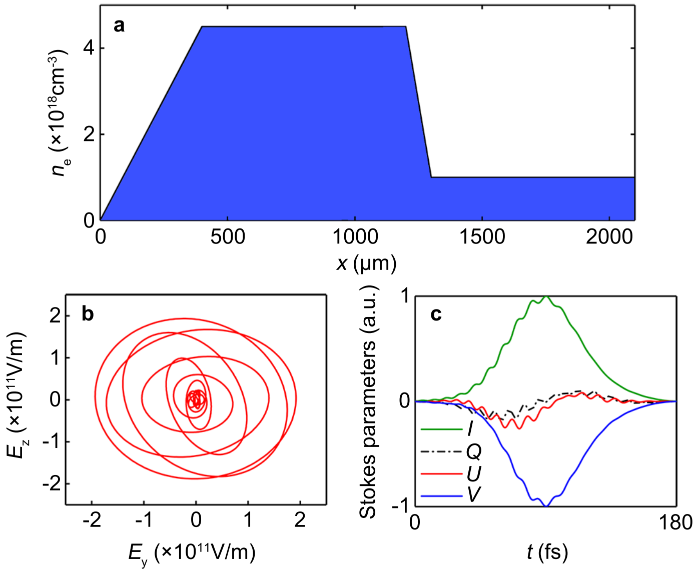

Fig. S4. The tailored plasma density structure and mid-IR polarization. a The tailored plasma density structure. **b** and **c** The Lissajous figure and the Stokes parameters of the on-axis mid-IR wave at $x = 1900$ μm with $B = 500$ T.

mid-IR pulse is left-handed, and the maximum intensity is $E_y \approx E_z \approx 1.8 \times 10^{11}$ V m^{-1}, respectively. The stokes parameters at the peak intensity position of the mid-IR pulse are $V \approx -1$, $Q \approx 0$, and $U \approx -0.06$ in Fig. S4c, which means that the mid-IR wave is nearly LHCP pulse. These results demonstrate that the plasma rotator still works at much lower intensity of external magnetic field in a tailored plasma.

5 The potential issues in simulations and experiments

To further illustrate the correctness of the simulation results and the operability of experiment, here, we demonstrate some potential issues by using 3D PIC simulations, including the spatially non-uniform external magnetic field, the transverse spatial resolution, the macroparticles per cell, the particles collision, the plasma temperature and the directed motion of the gas jet.

The first important issue in experiment is the practical external magnetic field required for the rotator. Taking the laser driven coil-target for example, the generated intense magnetic field is non-uniform spatially. In this case, the spatial distribution of the generated magnetic field conforms to the Biot-Savart Law, i.e., $B_x \sim R^2/(R^2 + x^2)^{3/2}$, where $R = 250$ μm is the radius of the coil. By using PIC simulations, we here demonstrated an example of the application of such magnetic field in our configuration, and the plasma density profile and the magnetic field distribution is shown in Fig. S5a, where the magnetic field B_x is generated by a laser with intensity of about 1.3×10^{16} W cm^{-2}, according to the Fig. 6 in Ref. 14. Figure S5b shows the spectral distribution of on-axis electric field at $x = 1700$ μm. It is demonstrated that the mid-IR pulse is generated with a center wavelength of about 5.2 μm and a spectral width of 4.1-8.8 μm. The Lissajous figures and the Stokes parameters of the on-axis mid-IR pulse are shown in Figs. S5c and S5d. It can be clearly seen that the mid-IR pulse is left-handed, and the maximum intensity is $E_y \approx E_z \approx 4.3 \times 10^{11}$ V m^{-1}, respectively. The Stokes parameters at the peak intensity position of

the mid-IR pulse are $V \approx -1$, $Q \approx -0.01$, and $U \approx -0.04$, which means that the mid-IR wave is nearly LHCP pulse. This demonstrated the robustness and feasibility of the proposed scenario in the practice.

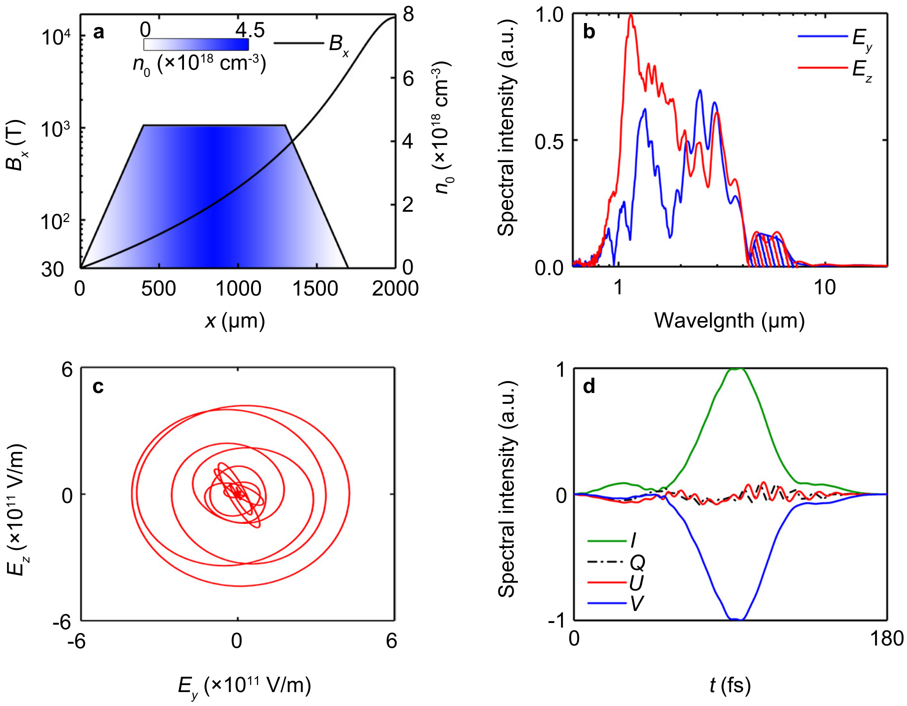

Fig. S5. The effect of plasma ejection on mid-IR polarizaiton. The spectral distribution of the on-axis electric filed (**a**), the Lissajous figure (**b**), and the Stokes parameters (**c**) for the plasma ejection with a sound speed.

The plasma can be produced by using a gas jet, as shown in Fig. 1, thus the effect of the directed motion of the gas jet should be considered. In general, the velocity of the plasma ejection from a gas jet is subsonic or supersonic. Thus, the directed motion velocity of the plasma ejection is about 3.4×10^{-4} μm ps^{-1}, and the propagation time of the relativistic laser in the magnetized plasma is about 5.6 ps. The movement distance of the plasma ejection during the interaction time is much less than 1 μm. Therefore, the directed motion of the plasma ejection does not affect the FVFR and the generation of the mid-IR pulse, which can be ignored in the simulations. In order to demonstrate this fact, we perform additional simulations by assuming a plasma ejection with a sound speed. The simulation results are shown in Fig. S6. One can see that the generated left-handed circular polarized mid-IR pulse (Stokes parameter $V_{min} \approx -0.96$) with a center wavelength of 6.7 μm, the spectral width of 5-25 μm, the electric field intensity of $E_{MIR} \approx 6.1 \times 10^{11}$ V m^{-1}, and the duration of 46.3 fs. The polarization state and beam parameters of mid-IR pulse generated are consistent with those in the main text. This indicates that the plasma ejection velocity in the current laboratories has limited influence on the final results.

The thermally expanded plasma can also be used to replace the gas jet in experiments, and the thermally expanded plasma can be readily prepared in a practical experiment. If the thermally expanded plasma is employed in our configuration, the plasma temperature effects should be considered, so that simulations have been carried out by assuming a plasma temperature of, e.g., 10 eV and 50 eV, respec-

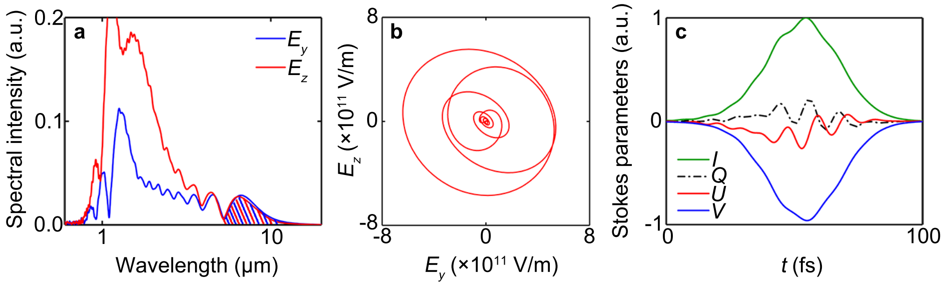

Fig. S6. The effect of plasma ejection on mid-IR polarizaiton. The spectral distribution of the on-axis electric filed (**a**), the Lissajous figure (**b**), and the Stokes parameters (**c**) for the plasma ejection with a sound speed.

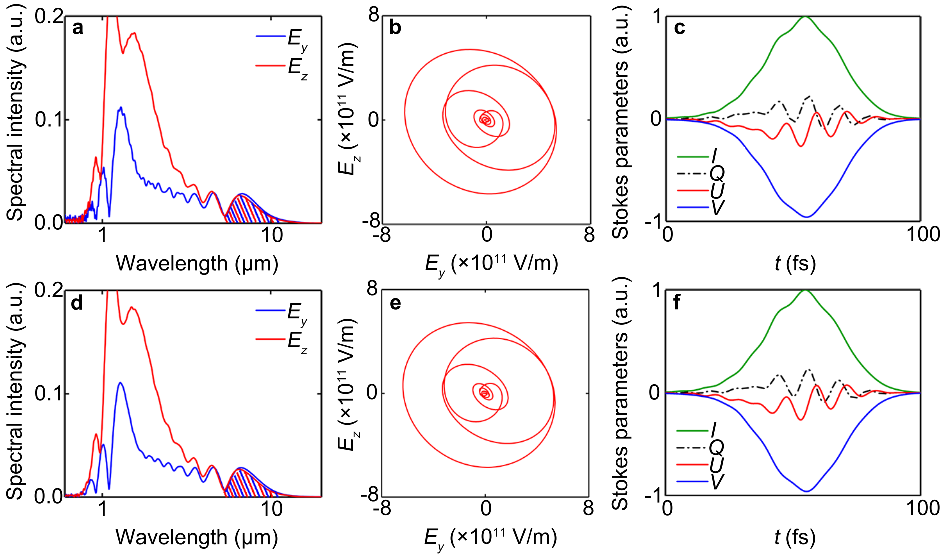

Fig. S7. The effect of plasma electron temperature on mid-IR polarizaiton. The spectral distribution of the on-axis electric field, the Lissajous figure, and the Stokes parameters for the thermally expanded plasma of 10 eV (**a-c**) and 50 eV (**d-f**), respectively.

tively, and the simulation results are presented in Fig. S7. As shown in Figs. S7a-S7c, one can see that a left-handed circularly polarized mid-IR pulse with Stokes parameter $V_{\mathrm{min}} \approx -0.96$, a center wavelength of 6.7 μm, a spectral width of 5-25 μm, an electric filed intensity of $E_{\mathrm{MIR}} \approx 6.0 \times 10^{11}$ V m^{-1} and a duration of 48.7 fs can be obtained at 10 eV. Similar results can be obtained at 50 eV, as shown in Figs. S7d-S7f. This also demonstrates the ignorable influences of thermal plasma effects on the final results.

For a relativistic plasma, the high-speed electron-ion collision may be significant. After the gas is ionized by the incident laser pulse, the electron temperature quickly reaches keV level, that is, the background plasma at the local location of the propagating laser is of high temperature but low density. Therefore, the particle collision time (under the condition in our simulations) is typically much greater than the laser pulse length, so that the collision frequency is very small. This means that the radiation from high-speed particle collisions is not particularly significant. In theory, the collision time of electrons and ions can be estimated by $\tau_{\mathrm{ei}} = (2\pi n_0 \lambda_{\mathrm{D}}^3)/(Z \ln \Lambda \omega_{\mathrm{p}})$ [15], where λ_{D} is the Debye length, Z is the atomic number, and $\ln \Lambda$ is the Coulomb logarithm. With our parameters detailed in the Methods section of

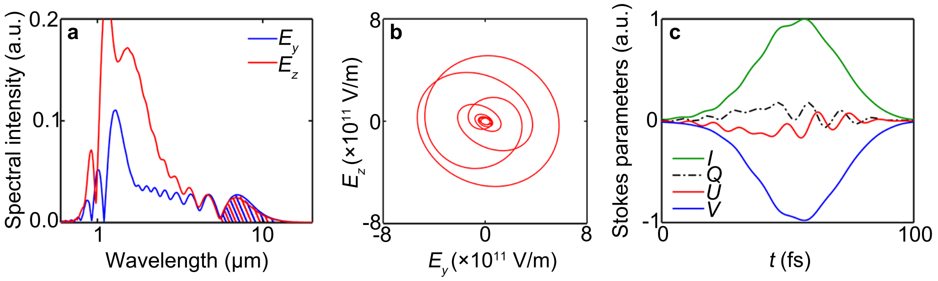

Fig. S8. The effect of electron-ion collisions on mid-IR polarizaiton. The spectral distribution of the on-axis electric field (**a**), the Lissajous figure (**b**), and the Stokes parameters (**c**) for the simulation with electron-ion collisions.

main text, the collision time of electrons and ions is $\tau_{\rm ei} \approx 3 \times 10^{-11}$ s, which is really much greater than the drive laser pulse duration. To demonstrate that the particle collisions do not severely affect the FVFR and the generation of mid-IR pulse, we perform additional simulations by considering the particle collisions. The results are shown in Fig. S8. One can see that the generated left-handed circular polarized mid-IR pulse is characterized by Stokes parameter $V_{\rm min} \approx -0.98$, a center wavelength of 7.0 μm, a spectral width of 5-25 μm, an electric filed intensities of $E_{\rm MIR} \approx 5.4 \times 10^{11}$ V m^{-1}, and a duration of 56.7 fs. These further demonstrate our conclusions above.

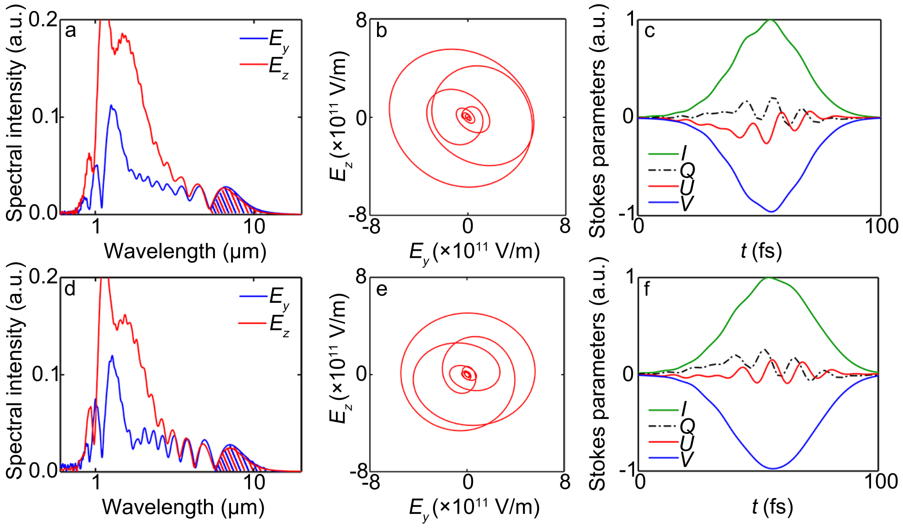

Fig. S9. The effect of grid number on mid-IR polarizaiton state. The spectral distribution of the on-axis electric field (**a**, **d**), the Lissajous figure (**b**, **e**), and the Stokes parameters (**c**, **f**) for 5 (top) and 10 (bottom) cells per drive laser wavelength in the transverse.

The transverse resolution is an important parameter in PIC simulations, which can impact the transverse evolution of the laser pulse, e.g., the laser focusing. To clarify the influences of the transverse resolution on the mid-IR pulse generation and manipulation, we have performed simulations by using 10 cells per transverse drive laser wavelength (twice as much as in the main text). Figure S9 shows the comparison of simulation results in two cases (5 and 10 cells). One can see that the generated mid-IR pulses have almost the same beam quality. For example, the generated mid-IR pulse with much higher transverse

resolution is still left-handed circularly polarized with Stokes parameter $V_{\min} \approx -0.98$, a center wavelength of 7.1 μm, the spectral width of 5.5-16.7 μm, and the electric filed intensity of $E_{MIR} \approx 5.4 \times 10^{11}$ V m^{-1}, and the duration of 55.4 fs, which is consistent with the low-resolution cases in the main text. This demonstrates that the transverse spatial resolution in the main text does not severely affect the laser evolution and plasma dynamics.

The macroparticles per cell is another important parameter in PIC simulations. In the main text, 2 macroparticles per cell as used in our simulations are acceptable, which can not change the underlying physics. Here, we present the additional simulation results using 4 and 8 macroparticles per cell in Fig. S10. The simulation results are consistent with the case of 2 macroparticles. Together with Fig. S9 above, we can conclude that the resulting simulation noise due to the limited transverse resolution and parcels per cell is relatively small, which does not severely affect the FVFR and the generation of mid-IR pulse.

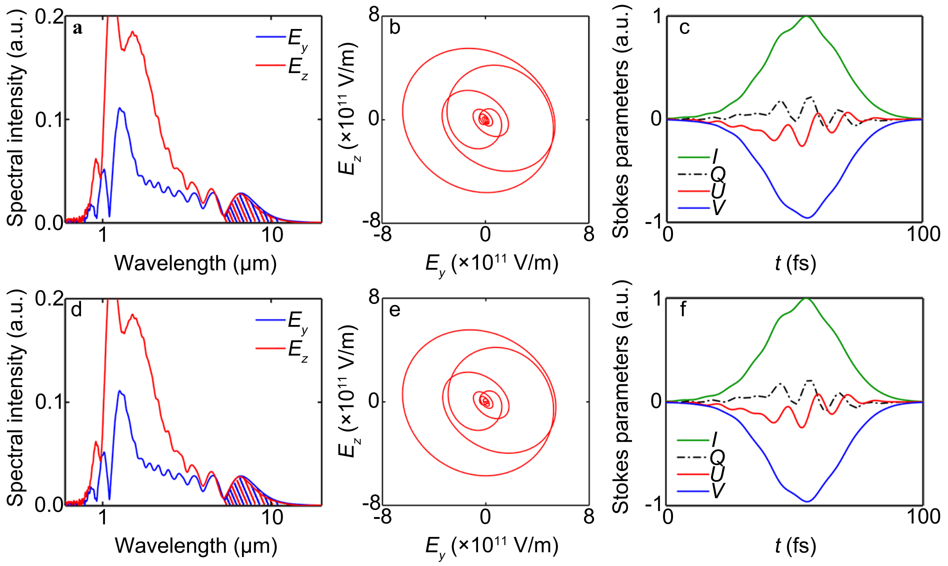

Fig. S10. The effect of macroparticles per cell on mid-IR polarizaiton state. The spectral distribution of the on-axis electric filed (**a**, **d**), the Lissajous figures (**b**, **e**), and the Stokes parameters (**c**, **f**) for 4 (top) and 8 (bottom) macroparticles per cell, respectively.

References

[1] Liang, Y., Sang, H.-B., Wan, F., Lv, C. Xie, B.-S. Relativistic laser pulse compression in magnetized plasmas. *Physics of Plasmas* **22**, 073105 (2015).

[2] Weng, S. M. et al. Extreme case of Faraday effect: magnetic splitting of ultrashort laser pulses in plasmas. *Optica* **4**, 1086–1091 (2017).

[3] Huang, Y.-C. & Dai, Z.-G. The Extreme Faraday Effect in Fast Radio Bursts. *The Astrophysical Journal Letters* **983**, L24 (2025).

[4] Ferrière, K., West, J. L. & Jaffe, T. R. The correct sense of Faraday rotation. *Monthly Notices of the Royal Astronomical Society* **507**, 4968–4982 (2021).

[5] Nie, Z. et al. Relativistic single-cycle tunable infrared pulses generated from a tailored plasma density structure. *Nature Photonics* **12**, 489–494 (2018).

[6] Nie, Z. et al. Photon deceleration in plasma wakes generates single-cycle relativistic tunable infrared pulses. *Nature Communications* **11**, 2787 (2020).

[7] Zhu, X.-L. et al. Efficient generation of relativistic near-single-cycle mid-infrared pulses in plasmas. *Light: Science & Applications* **9**, 46 (2020).

[8] Esarey, E., Schroeder, C. B. & Leemans, W. P. Physics of laser-driven plasma-based electron accelerators. *Reviews of Modern Physics* **81**, 1229–1285 (2009).

[9] Sprangle, P., Esarey, E. & Ting, A. Nonlinear theory of intense laser-plasma interactions. *Physics Review Letters* **64**, 2011–2014 (1990).

[10] Mori, W. B. The physics of the nonlinear optics of plasmas at relativistic intensities for short-pulse lasers. *IEEE Journal of Quantum Electronics* **33**, 1942-1953 (1997).

[11] Li, D. et al. Laser chirp controlled relativistic few-cycle mid-infrared pulse generation. *High Power Laser Science and Engineering* **11**, e57 (2023).

[12] Zhu, X.-L. et al. Generation of single-cycle relativistic infrared pulses at wavelengths above 20 µm from density-tailored plasmas. *Matter and Radiation Extremes* **7**, 014403 (2022).

[13] Sprangle, P., Esarey, E. & Ting, A. Nonlinear interaction of intense laser pulses in plasmas. *Physics Review A* **41**, 4463–4469 (1990).

[14] Fujioka, S. et al. Kilotesla magnetic field due to a capacitor-coil target driven by high power laser. *Scientific Reports* **3**, 1170 (2013).

[15] Takabe, H. The Physics of Laser Plasmas and Applications-Volume 1 (Cham: Springer, 2020).
